# Influence of Host-Related Factors and Exposure to Mosquito Bites on the Dynamics of Antibody Response to *Plasmodium falciparum* Antigens

**DOI:** 10.3390/tropicalmed6040185

**Published:** 2021-10-18

**Authors:** Kakou G. Aka, Serge S. Yao, Eric A. Gbessi, Akré M. Adja, Vincent Corbel, Alphonsine A. Koffi, Christophe Rogier, Serge B. Assi, Offianan A. Toure, Franck Remoue, Anne Poinsignon

**Affiliations:** 1 Institut Pierre Richet/Institut National de Santé Publique, Bouaké, Côte d’Ivoire; ghislain.aka@gmail.com (K.G.A.); adjamaurice@yahoo.fr (A.M.A.); koffi_alphonsine@yahoo.fr (A.A.K.); assisergi@yahoo.fr (S.B.A.); franck.remoue@ird.fr (F.R.); 2 MIVEGEC, University of Montpellier, IRD, CNRS, 34000 Montpellier, France; vincent.corbel@ird.fr; 3 Unité de Paludologie, Institut Pasteur de Côte d’Ivoire, Abidjan 01 BP 490, Côte d’Ivoire; yaoserstephane@gmail.com (S.S.Y.); ericadji@yahoo.fr (E.A.G.); andre_offianan@yahoo.fr (O.A.T.); 4 UFR Biosciences, University Felix Houphouet Boigny, Abidjan 01 BP V34, Côte d’Ivoire; 5 Primum Vitare!, Paris, France; christophe.rogier@gmail.com; 6 Institut Pasteur de Madagascar, Antananarivo 101, Madagascar; 7 Programme National de Lutte contre le Paludisme, Abidjan, Côte d’Ivoire

**Keywords:** *Plasmodium falciparum*, malaria, *Anopheles*, *Aedes*, humoral acquired immunity, saliva, immunomodulation, exposure

## Abstract

Humoral immunity to *Plasmodium falciparum* is acquired after repeated infections, and can lead to clinical protection. This study aimed to evaluate how human-, parasite-, and environment-related determinants can modulate the dynamics of IgG responses to *Plasmodium falciparum* after an infection. Individuals (*n* = 68, average age = 8.2 years) with uncomplicated malaria were treated with ACT and followed up for 42 days. IgG responses to *P. falciparum* merozoite antigens (*Pf*MSP1*, Pf*MSP3*, Pf*AMA1*, Pf*GLURP-R0), to whole schizont extract (*Pf*Schz), and to *Anopheles* gSG6-P1 and *Aedes* Nterm–34 kDa salivary peptides were measured. Regression analyses were used to identify factors that influence the dynamics of IgG response to *P. falciparum* antigen between D0 and D42, including demographic and biological factors and the level of exposure to mosquito bites. The dynamics of IgG response to *P. falciparum* differed according to the antigen. According to multivariate analysis, IgG responses to *Pf*Schz and to *Pf*GLURP-R0 appear to be affected by exposure to *Aedes* saliva and are associated with age, parasite density, and anti-*Plasmodium* pre-existing immune response at study inclusion. The present work shows that human exposure to *Aedes* saliva may contribute, in addition to other factors, to the regulation of anti-*Plasmodium* immune responses during a natural infection.

## 1. Introduction

In malaria-endemic settings, human populations acquire humoral immunity to *Plasmodium falciparum* (*P. falciparum*) after repeated infections, which can lead to clinical protection in reducing blood-stage parasitemia and life-threatening symptoms. Natural protective acquired immunity was first highlighted in studies where passive transfer of purified immunoglobulin G (IgG) obtained from malaria-immune adults successfully reduced *Plasmodium* parasitemia in children [1,2]. Although the targets and mechanisms of this protective immunity are not completely understood, several studies suggest that high antibody (Ab) levels against certain blood-stage merozoite antigens play an important role in clinical protection, making these *Plasmodium* antigens potential anti-malaria vaccine candidates. Clinical protective immunity is dependent on high concentrations of IgG as well as of cytophilic IgG1 and IgG3 Abs to blood-stage antigens, including apical membrane antigen 1 (*Pf*AMA1) [3,4], merozoite surface protein 1 and 3 (*Pf*MSP1, *Pf*MSP3) [5,6,7], and glutamate-rich protein (*Pf*GLURP-R0) [8]. However, recent study has found greater associations with protection when measuring functional antibodies that fix complement than antibodies that inhibit growth [9]. Longitudinal immunological studies have reported anti-*P. falciparum* Ab responses increase and peak within 1–2 weeks after a malaria infection, and then generally decline rapidly, suggesting a short-lived duration of circulating IgG to merozoite antigens (2–3 weeks) [10,11]. On the other hand, Ab responses may last for many years, and this persistence was positively associated with age, as a reflection of cumulative exposure to *Plasmodium* infections [12,13]. Immune responses are complex traits and may be influenced by different determinants in addition to intrinsic Ab half-life. A deeper understanding of host- and parasite-related factors, as well as of environmental factors, modulating the antigen-specific Ab response dynamics after a *Plasmodium* infection is important in selecting which antigens are valuable for the development of serological surveillance tools or anti-malaria vaccines.

Age, genetic factors, parasite density, previous exposure to *Plasmodium*, pathogen co-infection, and nutritional status are known to influence anti-*Plasmodium* Ab responses [14,15,16,17]. Environmental factors may also influence vertebrate immunity activity; annual seasonality is found to be an important environmental factor influencing cytokine production [18]. Immunomodulatory components of mosquito saliva are an interesting but often overlooked environmental factor.

In malaria-endemic areas, human populations are repeatedly exposed to salivary components of blood-feeding mosquitoes that possess a variety of pharmacologically active biomolecules with anti-hemostatic, anti-inflammatory, and immunomodulatory properties [19]. Mosquito immunomodulatory salivary proteins act both on innate and adaptive immunity [20], and studies suggested that T-cell populations are particularly susceptible to the effects of mosquito saliva [21,22]. T- and B-cell proliferation seems to be inhibited in a dose-dependent manner. High concentrations of saliva or repeated pre-exposure to mosquito saliva create an immunosuppressed environment, whereas decreasing saliva concentrations, instead, modulate the Th1/Th2 immune response [23,24,25,26]. A study examining immune responses to the bites of *An. stephensi* reported mast cell degranulation leading to local fluid and neutrophil influx and lymph node hyperplasia as a result of the recruitment of lymphocytes, dendritic cells, and monocytes [27]. *Anopheles* saliva also induced IL-10 in draining lymph nodes and downregulated antigen-specific T-cell responses [21]. Repeated pre-exposure to *Anopheles* bites also seems to skew the response towards that of the Th1 phenotype and to protect against *Plasmodium yoelii* infection [28], but these results are controversial [29,30]. Thus, human immune responses modulated by mosquito saliva are significant and complex in altering the frequencies of several immune cell populations and of cytokine production in multiple tissues, and can last for several days in the skin and at the systemic level [26].

However, there is presently only a limited understanding of the immune microenvironment initiated by arthropod salivary components in the vertebrate host and their role in the modulation of specific immunity against pathogens. A limited number of experimental studies showed that mice exposed to arthropod bites had a down-regulated antigen-specific immune response compared with naïve mice [21,31]. Studies in human populations living in malaria-endemic areas showed that acquired anti-*Plasmodium* Ab responses differed in children with varying exposure to *Anopheles* bites. A difference in the cytophilic Ab response to *Plasmodium* according to the intensity of exposure to *Anopheles* bites was reported, with a down-regulated IgG1 level seen in children with higher exposure, while IgG3 levels were similar in children with low or high exposure to *Anopheles* bites [32]. More recently, a study suggested that, in addition to other factors, *Anopheles* saliva may also down-modulate the anti-*Pf*MSP1 IgG, IgG1, and IgG3 immunity [33].

The major aims of the present study were to explore the key human-, parasite-, and environment-related factors that modulate anti-*Plasmodium falciparum* Ab dynamics after an infection. To address these aims, individuals with an uncomplicated clinical malaria attack visiting a health center in Bouaké (Côte d’Ivoire) were enrolled in the study, treated with artemisinin-based combination therapy (ACT), and followed up for 42 days. The variation in the anti-*Plasmodium* IgG response during follow-up and the identification of factors that may influence its dynamics were assessed through univariate and multivariate regression analysis. *Plasmodium* merozoite-stage specific antigens (*Pf*MSP1, *Pf*MSP3, *Pf*AMA1, and *Pf*GLURP-R0) and schizont extract (*Pf*Schz) were selected for their differences in immunogenicity and persistence [34,35] and for their potential in an advanced malaria vaccine [36].

The intensity of *Anopheles* and *Aedes* exposure was assessed at the individual level by evaluating the IgG response to the *Anopheles* gSG6-P1 and *Aedes* Nterm–34-kDa salivary peptides. During the last decade, these serological biomarkers were developed and aimed at evaluating the level of exposure to mosquito bites [37,38]. Specific human IgG against salivary peptides from *Anopheles* (gSG6-P1) and *Aedes* (Nterm–34 kDa) represents a proxy of human exposure to *Anopheles* and *Aedes* bites and is a reliable tool for assessing the spatial and temporal heterogeneity of exposure at the individual level [39,40].

## 2. Materials and Methods

### 2.1. Ethics Statement

The present study followed the ethical principles recommended by the Edinburgh revision of the Declaration of Helsinki and was approved by the Ethics Committee of the Côte d’Ivoire Ministry of Health (June 2014; No. 41/MSLS/CNER-dkn). Site leaders provided prior permission to survey on each site and written informed consent of all parents or guardians of children who participated in the study was obtained before inclusion.

### 2.2. Study Area

The study was conducted in Dar-es-Salam, a neighborhood of Bouaké city located in the center of Côte d’Ivoire, from April to June 2016. The local climate and malaria epidemiology have been previously described [33,41]. The area has intense malaria transmission and *P*. *falciparum* accounts for more than 95% of all human malaria infections [42], with *An. gambiae s.l.* being the major vector [43,44]. The climate is tropical humid with two seasons: The dry season runs from November through March, and the rainy season from April to October. Malaria transmission is perennial with seasonal upsurges during the rainy season.

### 2.3. Study Design, Procedure, and Sample Collection

The study was carried out during a clinical trial that aimed to assess the in vivo efficacy of artemisinin-based anti-malarial combination therapies. Details of the study, clinical procedures, and drug administration are described elsewhere [45]. Briefly, patients (*n* = 120, age > 6 months) with uncomplicated malaria and monospecific *P. falciparum* infestation confirmed by microscopy (parasite density between 2000 and 200,000 asexual parasites/μL of blood), axillary temperature of ≥37.5 °C, or a history of fever over the past 24 h, with body weight ≥5 kg, and who were able to take oral medications and follow study procedures were included in the study. All participants were treated with artemisinin-based combination therapies—artesunate + amodiaquine (AS + AQ) or artemether + lumefantrine (AL) —for 3 days (day 0 (D0), D1, D2), and then visited every week (between D7 and D42). Blood samples were collected repeatedly on D0, D3, and weekly from D7 to the end point of the follow-up (D42), for thick and thin blood smears. Dried blood spots (DBS) on Whatman 3 MM filter paper were collected on D0 and D42, air-dried and stored in plastic bags at +4 °C until immunological analysis. Thick blood smears were fixed and stained with 10% Giemsa and read double-blind by two certified microscopists; discordant readings were re-examined by a third qualified independent microscopist. Asexual parasite densities were counted against 200 microscope fields assuming 8000 white blood cells per microliter. A blood smear was considered negative if no parasites were observed.

The present study was carried out with the blood samples collected on D0 and D42 from 68 individuals out of the initial 120, including only individuals not lost to follow-up, without therapeutic failure, not becoming re-infected during the follow-up, and whose blood samples on D0 and D42 were available.

### 2.4. Parasite and Salivary Antigens

The *Plasmodium* merozoite-stage specific antigens (*Pf*MSP1, *Pf*MSP3, *Pf*AMA1, and *Pf*GLURP-R0) were produced in recombinant form and kindly provided by collaborators. *Pf*MSP-1p19 (Uganda-Palo-Alto strain) was expressed in a Baculovirus/insect cell system [46]; the recombinant *Pf*AMA1 (amino-acids 25–545, FVO strain) was expressed in *Pichia pastoris* [47]; and *Pf*MSP3 (amino acids 212–380, F32 strain) and *Pf*GLURP-R0 (amino acids 25–514, F32 strain) were expressed in *Escherichia coli* [48,49]. The schizont extracts (*Pf*Schz) were produced from *P. falciparum* cultures (strain 07/03, [50]). The gSG6-P1 and Nterm–34 kDa salivary peptides [51,52] were synthesized and purified (purity: >95%) by Genepep (St-Jean de Vedas, France). Peptides were shipped in lyophilized form and then resuspended separately in milliQ water and stored in aliquots at −20 °C until use.

### 2.5. Enzyme-Linked Immunosorbent Assay: Human Antibody Response to P. falciparum Antigens, gSG6-P1 and Nterm–34 kDa Salivary Peptides

Standardized dried blood spots (diameter: 1 cm) of each participant at two time points (D0 and D42) were eluted each in 350 μL phosphate buffered saline containing 0.1% Tween 20 (0.1% PBST) at +4 °C for 48 h. Human IgG levels against the gSG6-P1, Nterm–34 kDa, *Pf*Schz, *Pf*AMA1, *Pf*MSP1, *Pf*MSP3, and *Pf*GLURP-R0 antigens were measured by enzyme-linked immunosorbent assay (ELISA).

Subsequently, 96-well Maxisorp micro-assay plates (Nunc, Roskilde, Denmark) were coated overnight at 4 °C at a dilution of 1/1200 for *Pf*Schz, 1 µg/mL for *Pf*AMA1 and *Pf*MSP1, and 2 µg/mL for *Pf*MSP3 and *Pf*GLURP-R0. Plates were blocked with 5% skimmed milk powder (*w*/*v*) in 0.1% PBST for 1 h at room temperature. Individual eluates diluted in buffer (1% milk powder in 0.1% PBST) were added for incubation (2 h at room temperature) at a final dilution of 1/10 for *Pf*Schz, 1/50 for *Pf*MSP3 and *Pf*GLURP-R0, and 1/200 for *Pf*AMA1 and *Pf*MSP1. Plates were washed three times between each step with washing buffer (0.1% PBST). HRP-conjugated goat anti-human IgG (Frederick, USA), diluted at 1/1000 for *Pf*MSP3, 1/2500 for *Pf*GLURP-R0, and 1/5000 for all the other *P. falciparum* antigens, was added, and samples were incubated for 1 h at room temperature. After four further washes, TMB (Kementec, Taastrup, Denmark) was used as a substrate and the reaction was stopped by adding 0.2 M H_2_SO_4_ (100 µL/well). The optical density (OD) was read after 30 min at 450 nm. All samples were tested in duplicate, and if there was a discrepancy of greater than 25% between duplicates, the sample was retested. Individual results (ΔOD) are expressed as: ΔOD = ODx − ODn, where ODx represents the mean of the individual OD value in both wells with *P. falciparum* antigen and ODn the individual OD value for each eluate without antigen. A pool of positive hyperimmune serum collected from adult residents in a malaria-endemic area [32] was included on each plate to allow for standardization of day-to-day and plate-to-plate variation. Standard curves were established using human purified IgG protein (The Binding Site, Saint-Egreve, France) to convert ΔOD values for each sample into IgG concentrations (*CPf* in ng/mL). Each plate included a calibration (standard) curve with eight doubling dilutions of human purified IgG (3.12–400 ng/mL for schizont extract; 0.78–100 ng/mL for *Pf*AMA1 and *Pf*MSP1; 0.58–75 ng/mL for *Pf*GLURP-R0 and *Pf*MSP3). To construct the standard curve, we plotted the OD values at the corresponding known concentrations of purified human IgG, and then defined the standard curve using linear regression analysis. The IgG concentration of blood samples from participants were predicted from this equation based on the OD value. European nonimmune individual sera (*n* = 16, France) served as negative controls to determine the cut-off value for seropositivity for each *Plasmodium* antigen, defined as mean OD plus three standard deviations (mean[∆ODneg] +3 SDneg).

Serology testing of human exposure to mosquito salivary antigens (Nterm–34 kDa and gSG6-P1) was carried out by ELISA as described in [33] but with some modifications. Sera were incubated in duplicate wells at 4 °C overnight at a dilution of 1/40 in 0.1% PBST, mouse biotinylated Ab to human IgG (BD Pharmingen, San Diego CA, USA) was incubated at a dilution of 1/2000 in 1% PBST, and streptavidin biotin peroxidase was then added (1/1000; 1 h at 37 °C). Individual results are expressed as the ΔOD value: ΔOD = ODx-ODn, as mentioned above. Specific anti-gSG6-P1 and Nterm–34 kDa IgG levels were also assessed in European individuals (*n* = 16, France) to calculate the cut-off value for seropositivity for mosquito exposure (mean[∆ODneg] + 3 SDneg).

### 2.6. Immunological Data Analysis

The dynamics of specific IgG responses to *P. falciparum* antigens were investigated for each individual during the follow-up. Results are expressed as a ΔC*Pf* _D42-D0_ value, calculated according to the formula ΔC*Pf* _D42-D0_ = C*Pf*_D42_ − C*Pf*_D0_.

In the present study, we were interested in the effect of mosquito exposure on the evolution of humoral immunity to *Plasmodium* following an infection. Assuming that anti-salivary peptide IgG responses are transient and wane within a few weeks [53], evaluating the exposure on D42 may reflect exposure since current infection. Thus, individuals were separated into groups of exposure according to their IgG responses to the gSG6-P1 and Nterm–34 kDa peptides on D42. The mean values of the IgG responses to the salivary peptides were determined as the thresholds to define two groups of participants with different exposures (single-genus exposure) to *Anopheles* (lower exposure [LE_Ano_] < ΔOD_gSG6-P1_ = 1.41 < higher exposure [HE_Ano_]) and two groups with different exposures to *Aedes* (lower exposure [LE_Ae_] < ΔOD_Nterm-34 kDa_ = 1.10 < higher exposure [HE_Ae_]). Then, from these four groups of single-genus exposure, three exposure groups (combined *Anopheles* and *Aedes* exposure) were defined as follows: individuals with lower exposure to both *Anopheles* and *Aedes* were included in group E2 (Low); individuals with higher exposure to both *Anopheles* and *Aedes* were included in group E4 (High); and individuals from the lower-exposure group to one mosquito genus and from the higher-exposure group to the other mosquito genus were included in group E3 (Intermediate).

### 2.7. Covariates

Individual characteristics were analyzed as categorical (gender, ACT treatment) or continuous (age, weight, hemoglobin) variables to estimate their influence on the dynamics of Ab responses. Parasite density and immunologic responses (anti-*Plasmodium* IgG concentration on D0 and anti-salivary peptide IgG levels on D42) were analyzed as continuous variables in the linear analyses.

### 2.8. Statistical Analysis

Data generated from assays in the form of ∆OD values were entered into Microsoft Excel worksheets, and raw ∆OD representing IgG responses to *P. falciparum* were converted into concentration (C*Pf* ng/mL). All statistical analyses were conducted in R (Version 3.3.3; R Core Team, Vienna, Austria) with “tidyverse lattice” and “moment” packages. Figures were generated in R using the “ggplot2” package. A cut-off for seropositivity was determined for each antigen, and individuals were categorized as seropositive if their Ab response was above the cut-off value.

As immunologic data were not normally distributed, nonparametric tests were used for statistical analyses. The Wilcoxon signed-rank test was used to examine individual differences in Ab during the follow-up, the Mann–Whitney U (Wilcoxon rank-sum) test was run for comparison of Ab levels between two independent groups, and the Kruskal–Wallis test was used for differences among more than two groups. The association between two continuous variables was determined through Spearman’s correlation coefficient (Spearman’s rho, and *p* values are reported).

Multivariate linear regression models were then used for all covariates with a *p*-value of < 0.20 in univariate analysis. Final models were adjusted by backward selection and by removing non-significant variables of *p* > 0.05. Maximum likelihood method tests were used to identify the best-fitting models according to the Akaike information criterion (AIC) value. All differences were considered significant at *p* < 0.05.

## 3. Results

### 3.1. Population Baseline Characteristics

A total of 68 individuals were included in this study: 41 (60.3%) females and 27 (39.7%) males with an average age of 8.2 years (95% confidence interval (CI), (6.9–9.5)). The age structure of the population was 22 children ≤5 years, 24 children between 6 and 9 years of age, and 22 participants ≥10 years. The overall mean *P. falciparum* density at study inclusion was 54,965 parasites/µL (95% CI, (42,673–70,798)), and there was no difference in parasite density between age groups (*p* = 0.369). The mean hemoglobin (Hb) concentration was 12.38 g/dL and 11.82 g/dL, respectively, on D0 and D42 (*p* = 0.665).

### 3.2. Human IgG Response to P. falciparum and Mosquito Salivary Antigens during Follow-Up

The concentration levels of IgG to *P. falciparum* antigens (ng/mL) and to mosquito salivary peptides (in OD value), as well as seroprevalence both at the time of inclusion (D0) and at the end of the follow-up (D42), are presented in Table 1.

On D0, most of the individuals had a specific IgG response to *P. falciparum* antigens, ranging from 61.8% for *Pf*MSP1 to 100% for *Pf*Schz. For all merozoite antigens, the seroprevalence decreased during the follow-up, except for *Pf*AMA1, for which a similar rate was noted between D0 and D42. All individuals had a specific IgG response to *Pf*Schz at the two time points.

A wide range of anti-*Pf*AMA1, anti-*Pf*MSP1, and anti-*Pf*Schz IgG concentration levels on D0 and D42 were observed in individuals, whereas other anti-*Plasmodium* IgG responses were much more limited. Anti-*Pf*Schz IgG median levels increased significantly during the follow-up (*p* < 0.0001), whereas participants had significantly lower anti-*Pf*GLURP-R0 IgG median levels at the end of the follow-up (*p* < 0.001). No significant differences were noted in the median levels of IgG responses to *Pf*AMA1, *Pf*MSP1, and *Pf*MSP3 between D0 and D42.

We also evaluated the individual level of exposure to *Anopheles* and *Aedes* bites at the two time points by assessing the IgG response to the *Anopheles* gSG6-P1 and *Aedes* Nterm–34 kDa salivary peptides. The seroprevalence of anti-salivary peptide IgG was 100% and the wide range of ΔOD _gSG6-P1_ and ΔOD _Nterm-34 kDa_ values indicated that all participants were exposed to mosquito bites, albeit at different levels of exposure. However, no differences in the IgG median levels were noted between D0 and D42 (Wilcoxon test, *p* > 0.05) for the two salivary antigens, indicating a similar exposure to *Anopheles* and *Aedes* bites before (exposure estimated on D0) and during the cohort follow-up (exposure estimated on D42).

### 3.3. Dynamics of IgG Responses to P. falciparum According to Anopheles and Aedes Exposure

We assessed the evolution of the concentration of IgG against *P. falciparum* antigens (ΔC*Pf* _D42-D0_) in participants according to their individual levels of exposure to *Anopheles* and *Aedes* bites. Exposure was considered first at a single-genus level (*Anopheles* [LE_Ano_ and HE_Ano_] or *Aedes* [LE_Ae_ and HE_Ae_] separately) and, secondly, at a combined level (*Anopheles* and A*edes* taken together: E2 = low-, E3 = intermediate-, E4 = high-exposure groups).

**Figure 1:**
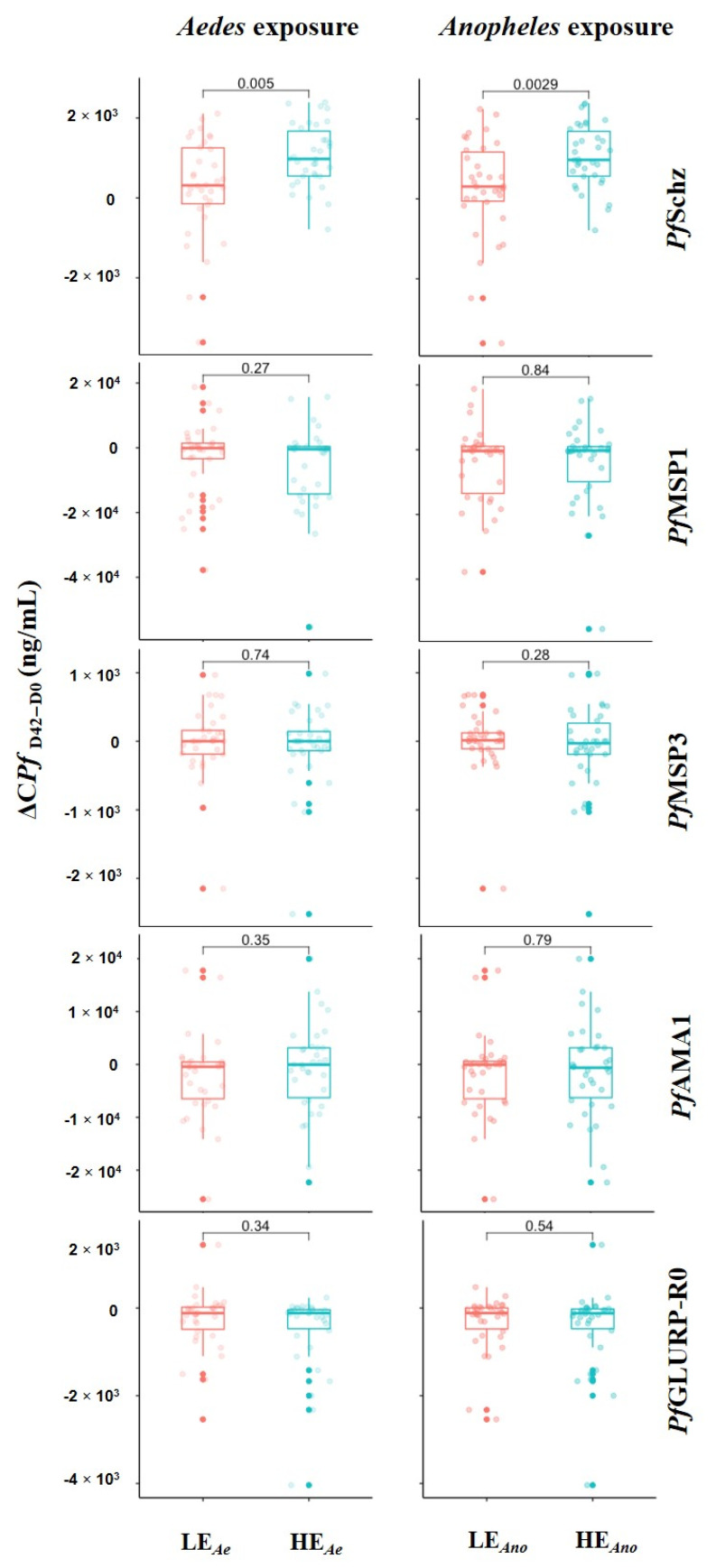
Variation in concentration of IgG response to *P. falciparum* antigens according to group of exposure to *Anopheles* and to *Aedes* (single-genus exposure). Participants were grouped according to their level of IgG response to *Anopheles* (lower exposure (LEAno) < ΔODgSG6-P1 = 1.41 < higher -exposure (HEAno)) and to *Aedes* (lower exposure (LEAe) < ΔODNterm-34 kDa = 1.10 < higher exposure (HEAe)) salivary peptide. Each dot represents an individual, and box plots indicate the median values, 25th and 75th percentile antibody concentration for each *P. falciparum* blood-stage antigen. Statistically significant difference in median value between the two groups is indicated (Mann–Whitney U test).

First, we compared the dynamics of IgG responses to *Plasmodium* antigens (ΔC*Pf* _D42-D0_) between the groups with lower and higher exposure to *Anopheles* or *Aedes* bites (Figure 1). The dynamics of immune responses were calculated as the concentration at D42 minus the concentration at D0, which meant that the median values close to zero showed similar response levels between the 2 time points, not zero antibodies.

Individuals who had a higher exposure to *Anopheles* (HE_Ano_) or to *Aedes* (HE_Ae_) bites exhibited a greater increase in anti-*Pf*Schz IgG levels than those with a lower exposure (*p* = 0.0029 for *Anopheles* and *p* = 0.005 for *Aedes*). No significant differences in the variation of IgG responses to merozoite antigens were noted between the two groups exposed to *Anopheles* or *Aedes* bites.

**Figure 2:**
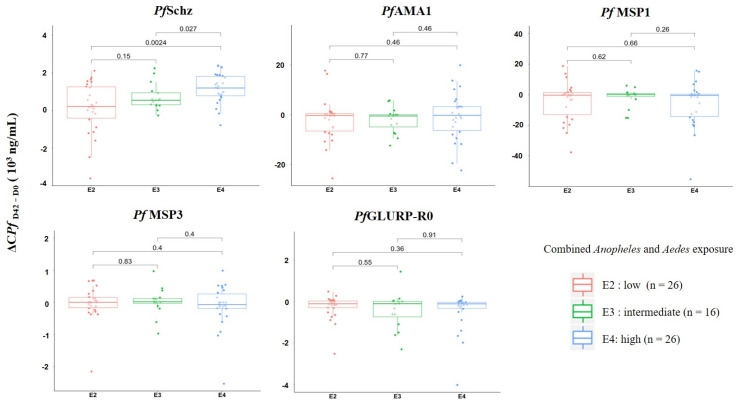
Variation in concentration of IgG response to *P. falciparum* antigens according to group of exposure to *Anopheles* and to *Aedes* (combined exposure). Each dot represents an individual, and box plots indicate the median value, 25th and 75th percentile antibody concentration for each blood-stage antigen. Individuals were divided into three groups (E2, E3, E4) where E2 includes individuals with lower exposure to both *Anopheles* and *Aedes,* E4 includes individuals with higher exposure to both *Anopheles* and *Aedes*, and E3 includes individuals with weak exposure to one mosquito genus and high exposure to the other. Statistically significant difference in median value between two groups is indicated (Mann–Whitney U test).

Second, we compared the dynamics of anti-*Plasmodium* immune responses according to the level of combined exposure to both *Anopheles* and *Aedes* bites (E2 = low, E3 = intermediate, E4 = high; Figure 2). We observed a positive association between the increase in the IgG response to *Pf*Schz and the level of mosquito exposure during the follow-up. Indeed, individuals with higher exposure to *Anopheles* and *Aedes* (E4 group) had a significantly greater increase in anti-*Pf*Schz IgG response compared with individuals from the group with intermediate exposure (E3 group, *p* = 0.027) and from the group with lower exposure (E2 group, *p* = 0.0024). No significant differences in the dynamics of anti-merozoite antigen IgG responses were noted between the exposure groups, with all median values close to null for any exposure group (combined exposure).

### 3.4. Human-, Parasite-, and Environment-Related Factors Associated with the Evolution of Immune Responses to Plasmodium

Univariate analyses were first performed to investigate the relationship between the individual variation in specific anti-*Plasmodium* IgG responses and different demographic, biological, parasitological, and environmental variables (Table 2).

Gender, hemoglobin concentration, and parasite density on D0 were not associated with any variation in IgG response to *Plasmodium* antigens (*p* > 0.05). The type of ACT treatment was also not associated with any variation in immune responses to *Plasmodium* antigens except for *Pf*MSP3 (*p* = 0.021), while age and weight were associated with variations in IgG against *Pf*Schz (*p* = 0.049 and *p* = 0.037, respectively) and *Pf*AMA1 (*p* = 0.051 and *p* = 0.006, respectively). In addition, variations in IgG against all antigens were negatively associated with the initial concentration on D0 (*p* < 0.002). We also assessed the effect of exposure to *Anopheles* or *Aedes* bites (continuous variable) on the variation in the specific anti-*Plasmodium* IgG responses. Only the anti-*Pf*Schz IgG level was positively associated with the level of exposure to *Anopheles* (*R* = 0.405, *p* < 0.001) and *Aedes* (*R* = 0.389, *p* = 0.001) bites. Other specific IgG response variations were not associated with the intensity of exposure to mosquito bites, although there was a borderline correlation between anti-*Pf*GLURP-R0 IgG and *Aedes* exposure (*R* = −0.228, *p* = 0.06).

### 3.5. Multivariate Analysis: Final Models

To further understand the contribution of each factor (confounding variables) to the evolution of anti-*Pf*Schz and anti-*Pf*GLURP-R0 IgG responses, multivariate analyses were carried out, and the final models are shown in Table 3. Multivariate analyses showed a positive and significant relationship between age and variation in IgG response to *Pf*Schz and *Pf*GLURP-R0, while no relationships were noted for weight. The level of *P. falciparum* parasite density on D0 was only associated with an increase in anti-*Pf*Schz IgG level. The models also showed that the variation in IgG responses to *Pf*Schz and *Pf*GLURP-R0 was strongly and negatively correlated with the pre-existing IgG level at D0 (*p* < 0.01). Individuals with higher specific IgG levels at the time of inclusion had lower levels on D42. Interestingly, a strong association was detected between the level of exposure to *Aedes* bites and the variation in anti-*Pf*Schz (*p* < 0.001) and anti-*Pf*GLURP-R0 (*p* < 0.05) IgG response, while *Anopheles* exposure was not found to be associated with the evolution of IgG response to *P*. *falciparum* antigens.

## 4. Discussion

In the present study, we described the variation in anti-*Plasmodium* IgG responses from study inclusion (D0) to the end of follow-up (D42) in relation to factors that might influence the kinetics of specific Ab responses. To exclude the antigenic boost of a *P. falciparum* infection on the immune responses examined, only individuals who were not re-infected were included in the final analysis. Multivariate analysis showed that age, parasite density (only for schizont extracts), anti-*Plasmodium* IgG response on the day of malaria diagnosis, and exposure to *Aedes* saliva were significantly associated with the variation in anti-*Plasmodium* IgG responses.

At the time of inclusion, a high inter-individual variability in IgG responses was observed for *Pf*AMA1, *Pf*MSP1, and *Pf*Schz antigens, while a lower level and range of IgG responses were seen for the other antigens, highlighting the difference in immunogenicity against *Plasmodium* antigens [35]. During the 6-week follow-up period, the evolution of the prevalence and median values of IgG specific to *P. falciparum* differed depending on the merozoite antigens. We observed a significant increase in IgG responses to the schizont extract and a significant decrease in anti-*Pf*GLURP-R0 IgG responses, while similar levels of IgG responses were noted for the other merozoite antigens. Longitudinal immunological studies have reported that following a clinical malaria infection, the specific IgG responses peak approximately 1–2 weeks after infection and then generally decline rapidly because of the short-lived duration of natural IgG responses to merozoite antigens [10,11,12,13,54,55]. It has also been shown that IgG Ab responses have a different half-life depending on the antigens [56], and this could contribute to the differences in the dynamics of immune responses to the various *Plasmodium* antigens observed during the follow-up. Parasite- and host-related or environmental factors may also play a role, in addition to intrinsic antibody half-life, in the modulation of the IgG response to *P. falciparum* over time.

We used an innovative serological assessment method to define a proxy of human exposure to *Anopheles* and *Aedes* bites at the individual level for each participant. This approach takes into account the individual heterogeneity of exposure to mosquitoes. Indeed, environmental factors generating hot spots of exposure (e.g., proximity to breeding sites), the attraction an individual exerts on mosquitoes, and the use of personal protection against mosquito bites (nets, coils, etc.) suggest that exposure to mosquito bites is highly variable from house to house and also between people living in the same house. The relevance of the gSG6-P1 and Nterm–34 kDa biomarker for epidemiological studies has been validated in various settings [37,40,41,57,58]. At the two time points of the follow-up, all of the participants had specific IgG to salivary peptides, indicating that they had all been exposed to *Anopheles* and *Aedes* bites before and during the follow-up, with an inter-individual heterogeneity in intensity of responses. According to their individual anti-gSG6-P1 and anti-Nterm–34 kDa IgG levels, individuals were separated into different groups of exposure according to a single-genus exposure and to combined exposure. Assuming that anti-salivary peptide IgG responses are transient and wane within a few weeks without new exposure [53,59], evaluating the exposure on D42 may reflect exposure since study initiation.

We first compared the dynamics of IgG titers against *P. falciparum* antigens between the different exposure groups both at the single-genus and at the combined-exposure level. We noted a positive association between the evolution of IgG responses to whole schizont extract and the intensity of exposure to *Culicidae* bites. Individuals with a higher exposure to *Anopheles* or *Aedes* (considered at the single-exposure level), as well as taken together (combined exposure), had a significantly increased IgG response to *Pf*Schz during the 6-week follow-up. The evolution of IgG responses to merozoite antigens did not seem to be modulated by the intensity of mosquito exposure.

The contribution of immunomodulatory components of mosquito saliva in the variation of anti-*Pf*Schz and anti-*Pf*GLURP-R0 IgG responses was then assessed using univariate analysis complete with multivariate models taking into account other co-factors. Among the different factors selected, gender, weight, hemoglobin concentration, and ACT treatment were not significantly associated with variations in IgG response to *Pf*Schz and to *Pf*GLURP-R0 during the follow-up.

The evolution of IgG responses to *Pf*Schz and to *Pf*GLURP-R0 had a strong positive association with the age of the individual. Indeed, in general, antibody levels increase with both age and higher transmission intensity [60,61]. History of infection and cumulative malaria exposure may contribute to the inter-individual differences in the dynamics of immune responses [15]. Older individuals may have experienced more malaria infections and thus may have developed more memory B cells that will rapidly proliferate upon reinfection and differentiate into long-lived antibody-secreting cells that may last longer and induce a greater magnitude and longevity of IgG response to antigens [10,13]. Nevertheless, in the present study, most participants were under 10 years old, thus limiting the comparison with older people, and therefore, further investigations are needed. The parasite density at the time of diagnosis was significantly positively associated with the evolution of IgG response to schizont extracts. It can be expected that a higher parasite load will induce a greater immune response to *Plasmodium* parasites [12]. Interestingly, the initial level of IgG response to *Plasmodium* merozoite antigens (*PfSchz*, *Pf*GLURP-R0) at the time of diagnosis was negatively associated with the variation in immune responses during the follow-up. This result indicates that participants with higher levels of Abs at the time of diagnosis might produce lower levels of IgG specific to *Plasmodium*. In malaria infection, natural regulatory T cells and positive/negative regulators have been shown to play a role in balancing immune responses to maintain vertebrate defense and immune balance. *Plasmodium* exploits regulatory mechanisms to prevent the development of adaptive immunity by enhancing negative regulators and/or inhibiting positive regulators including reduced T-cell proliferative responses to *Plasmodium* antigens and production of immunosuppressive IL-10 [62]. Negative regulators such as BTLA have been reported to dampen innate and T-/B-cell-mediated immune response to malaria infection [63,64]. However, antibodies measured at inclusion (D0) might have been acquired during previous infections but also by the current one. Indeed, there is obviously a delay between the infection and onset of symptoms, and then the visit to a health center, during which immune responses to *Plasmodium* are mounted.

The exposure to *Aedes* bites was associated with the evolution of IgG response to *Pf*Schz and *Pf*GLURP-R0, while *Anopheles* exposure was not found to modulate anti-*Plasmodium* immunity in the 6-week follow-up period. It is increasingly recognized that the immune modulatory properties of mosquito saliva act both on the innate and adaptive immune responses of the vertebrate host, but only a few studies have assessed the consequences of *Plasmodium* immune responses in a natural population [14,32,33,65]. These studies investigated the immune relationship within a homologous malaria context (*Anopheles*–*Plasmodium*), while in the present study, we observed different anti-*Plasmodium* immunity modulation according to the mosquito genus with a significant effect of *Aedes* exposure. Immunomodulatory properties of mosquito saliva on the specific immune responses to heterologous antigens have also been observed in murine studies [21,31]. Previous work indicated that *Culicidae* saliva has different immunomodulatory effects on the vertebrate immune system depending on the mosquito genus. Wanasen et al. suggested that the production of both Th1 and Th2 cytokines was reduced in the presence of salivary gland extract from *Aedes aegypti*, but not from *Culex quinquefasciatus* [22]. Mosquito saliva is known to contain close to 100 secretory proteins, and comparative analyses indicated that some salivary proteins are ubiquitous in various genera and/or species, while others are species or genus specific [66,67]. This suggests that mosquito saliva from various mosquito species should have common effects as well as genus- or species-specific immunomodulatory effects. Here, compared with *Anopheles* saliva, the immunomodulatory activities of *Aedes* saliva on anti-*Plasmodium* immunity seemed to be more pronounced. *Aedes* saliva has been investigated extensively and studies reported a profound effect on lymphocyte and macrophage activity [68,69]. In the present study, we showed that *Aedes* exposure was negatively associated with the anti-*Pf*GLURP-R0 immune response, whereas it was positively associated with the anti-*Pf*Schz IgG response. The epidemiological observation for the down-regulated IgG response to *Pf*GLURP-R0 in individuals with higher exposure is consistent with previous studies that reported a down-regulated immune response to a specific antigen in hosts exposed to arthropod saliva compared with naive vertebrate hosts [21,32,33]. Schizonts comprise a set of antigens with varying immunogenicity, while *Pf*GLURP-R0 is a unique antigen at the surface of infected erythrocytes. A schizont may thus activate a higher number of immune cells (memory B cells or long-lived plasma cells), which may obviously result in a stronger IgG response that may last longer.

There are several important limitations of this study. First, the number and average age of the participants were low, limiting the statistical power of our analyses and limiting the comparison with immune response evolution in older individuals. The 42-day follow-up was too short to study the decrease in the IgG response after *Plasmodium* infection. We observed that most individuals exhibited very little variation in their IgG responses to certain merozoite antigens between D0 and D42. The peak of the immune response occurs approximately 2 weeks after infection and then gradually wanes. The 42-day follow-up may be the time when the level of immune response returns to its level at the time of inclusion. A more intensive and long-term sampling would be needed to track the decrease in Abs more accurately over a longer period; furthermore, the study should ideally assess the asymptomatic re-infection by *Plasmodium* during follow-up, which might cause a boost in immune responses.

The present work is the first to report that exposure to *Aedes* saliva may contribute, in addition to other factors, to the regulation of anti-*Plasmodium* immune responses. Additional studies are needed to characterize separately the effects of saliva of different blood-feeding arthropods on the human immune system, by analyzing ex vivo cytokine production after stimulation of peripheral blood mononuclear cells. The immunomodulatory properties of mosquito saliva and their consequences for malaria transmission need further investigation and may contribute to a better understanding of the human–vector–parasite immune relationships.

## Figures and Tables

**Table 1:** IgG seroprevalence and level of IgG response to *Plasmodium falciparum* antigens and to gSG6-P1 and Nterm–34 kDa salivary peptides on D0 and D42.

	D0		D42		*p* Value ^b^
	IgG Prevalence ^a^ (%)	Median (IQR)	IgG Prevalence (%)	Median (IQR)	
*Pf*AMA1	70.58	5.78 (0.43–1.28)	73.52%	2.64 (0.70–9.53)	0.110
*Pf*GLURP-R0	70.58	0.20 (0.02–0.64)	17.65%	0.04 (0–0.12)	**<0.001**
*Pf*MSP1	61.76	1.95 (0.30–25.0)	51.47%	2.27 (0.40–9.65)	0.541
*Pf*MSP3	82.35	0.13 (0–0.37)	61.76%	0.12 (0–0.50)	0.987
*Pf*Schz	100	1.15 (0.78–1.76)	100%	2.09 (1.31–2.85)	**<0.0001**
gSG6-P1	100	1.32 (1.12–1.60)	100%	1.41 (1.07–1.61)	0.760
Nterm–34 kDa	100	1.06 (0.91–1.25)	100%	1.1 (0.86–1.31)	0.638

IQR: Interquartile range. Median values of IgG concentration to *P. falciparum* antigens expressed at 10–3 ng/µL. ^a^ European nonimmune individual sera (*n* = 16, France) served as negative controls to determine the cut-off value for seropositivity for each *Plasmodium* antigen, defined as mean OD plus three standard deviations (mean[∆ODneg] + 3 SDneg). ^b^ *p* value for the comparison of the antibody levels between D0 and D42 for each antigen, determined by Wilcoxon rank-sum test.

**Table 2:** Influence of covariates on the dynamics of IgG antibody levels to various *Plasmodium* and mosquito salivary antigens.

Covariate Factors	Antigens	ΔC*Pf* _D42−D0_ (ng/mL)	*p* Value
Gender (female/male) ^a^	*Pf*Schz	968.6 (178.2;1544)/561.3 (92.68;1213)	0.250
	*Pf*AMA1	−1151 (−6045;525.5)/413.2 (−9406;3340)	0.322
	*Pf*MSP1	−480.2 (−14,723;1322)/−267 (−3129;442.8)	0.744
	*Pf*MSP3	0 (−165.8;154.5)/0 (−181.5;300.1)	0.970
	*Pf*GLURP-R0	−109.6(−349.6;17.33)/−118.8(−750.8;0)	0.408
Age (years) ^b^	*Pf*Schz	0.240	**0.049**
	*Pf*AMA1	−0.013	**0.051**
	*Pf*MSP1	−0.208	0.088
	*Pf*MSP3	0.055	0.653
	*Pf*GLURP-R0	−0.162	0.186
Weight (kg) ^b^	*Pf*Schz	0.252	**0.037**
	*Pf*AMA1	0.405	**0.006**
	*Pf*MSP1	−0.128	0.298
	*Pf*MSP3	0.084	0.495
	*Pf*GLURP-R0	−0.173	0.158
Hemoglobin (D42) ^b^	*Pf*Schz	−0.123	0.330
	*Pf*AMA1	−0.125	0.317
	*Pf*MSP1	−0.112	0.273
	*Pf*MSP3	−0.141	0.261
	*Pf*GLURP-R0	−0.344	0.061
Treatment (ASAQ/AL) ^a^	*Pf*Schz	725.2 (178.2;1544)/704.3 (92.68;1213)	0.840
	*Pf*AMA1	−224.0 (−6045;525.5)/−793.9 (−9406;3340)	0.888
	*Pf*MSP1	−414.6 (−14,723;1322)/−237.0 (−3129;442.8)	0.577
	*Pf*MSP3	−100.2 (−165.8;154.5)/20.53 (−181.5;300.1)	**0.021**
	*Pf*GLURP-R0	−152.7 (−349.6;17.33)/−92.63 (−750.8;0)	0.535
Parasite density on D0 ^b^	*Pf*Schz	0.176	0.149
	*Pf*AMA1	0.096	0.433
	*Pf*MSP1	0.050	0.692
	*Pf*MSP3	0.053	0.670
	*Pf*GLURP-R0	0.036	0.768
IgG concentration on D0 (ng/mL) ^b^	*Pf*Schz	−0.361	**0.002**
	*Pf*AMA1	−0.360	**0.002**
	*Pf*MSP1	−0.625	**<0.001**
	*Pf*MSP3	−0.540	**<0.001**
	*Pf*GLURP-R0	−0.851	**<0.001**
anti-gSG6-P1 IgG ^b^*(Anopheles* exposure)	*Pf*Schz	0.405	**<0.001**
	*Pf*AMA1	−0.069	0.574
	*Pf*MSP1	−0.043	0.727
	*Pf*MSP3	−0.060	0.623
	*Pf*GLURP-R0	−0.129	0.294
anti Nterm-34 kDaIgG ^b^*(Aedes* exposure)	*Pf*Schz	0.389	**0.001**
	*Pf*AMA1	0.055	0.652
	*Pf*MSP1	−0.164	0.180
	*Pf*MSP3	−0.140	0.251
	*Pf*GLURP-R0	−0.228	0.060

^a^: Median (25th; 75th percentile) of variation of IgG to *P. falciparum* antigens during the follow-up. ^b^: Spearman’s rho.

**Table 3:** Results of multivariate analysis indicating factors associated with variation in IgG responses to *P. falciparum* antigens during the follow-up.

Variation in Anti-*Pf*Schz IgG					Variation in Anti-*Pf*GLURP−R0 IgG				
Variables	β1	Sd Error	*p* Value	*R^2^*	Variables	β1	Sd Error	*p* Value	*R^2^*
				0.60					0.81
Age	49.30	16.32	<0.01		Age	29.723	7.566	<0.01	
Weight	-	-	-		Weight	-	-	-	
Parasite density on D0	0.004	0.001	<0.001		Hemoglobin	-	-	-	
Anti-*Pf*SchzIgG on D0 (ng/mL)	−0.753	0.100	<0.001		Anti-*Pf*GLURP-R0 IgG on D0 (ng/mL)	−0.806	0.049	<0.01	
Anti−Nterm-34 kDa IgG	1185	277.3	<0.001		Anti-Nterm-34 kDa IgG	−276.11	132.261	<0.05	
Anti-gSG6-P1 IgG	-	-	-						

## Data Availability

The dataset analyzed during the current study is available from the last author on reasonable request.
